# Assessment of psychometric properties and measurement invariance of the sensation seeking scale for children in a Norwegian sample

**DOI:** 10.3389/fpsyg.2024.1341609

**Published:** 2024-02-16

**Authors:** Ellen Beate Hansen Sandseter, Ole Johan Sando, Rasmus Kleppe, Håvard Lorås, Lise Storli

**Affiliations:** ^1^Department of Physical Education and Health, Queen Maud University College of Early Childhood Education, Trondheim, Norway; ^2^Department of Teacher Education, Faculty of Social and Educational Sciences, Norwegian University of Science and Technology, Trondheim, Norway

**Keywords:** internal consistency, factor structure, measurement invariance, age, gender

## Abstract

**Introduction:**

The aim of this study was to examine the psychometric properties of a Norwegian translation of the Sensation Seeking Scale for Children (SSSC), designed for children between 7 and 12 years of age.

**Methods:**

A sample of 393 children (7–10 years old) were recruited to participate in the study. The SSSC was administered through interviews with each child, wherein their responses to the SSSC questionnaire were recorded on a tablet.

**Results:**

Analysis of internal consistency reliability did not show acceptable reliability for all subscales, and confirmatory factor analysis (CFA) showed that the Thrill Seeking and Behavioral Intensity subscales were associated and somewhat overlapping, while Behavioral Inhibition appeared as a single factor. A further explanatory factor analysis (EFA) revealed a two-factor solution. CFA of the two-factor solution resulted in the removal of some items in both factors due to low factor loadings. The final factors resulting from this analysis were Thrill and Intensity Seeking (13 items) and Behavioral Inhibition (7 items). The results also show that boys scored higher than girls on Thrill and Intensity Seeking while girls scored higher than boys on Behavioral Inhibition. Furthermore, age was positively associated with both subscales, meaning that older children tended to score higher.

**Discussion:**

The results in this study suggest that measures of children’s sensation seeking might be sensitive to cultural contexts and that, at least in a Norwegian population, a two-factor solution of the SSSC is recommended.

## 1 Introduction

In several theories of individual differences in personality and temperament, the extraversion dimension includes a trait that is characterized by the willingness to take risks and seek excitement, thrill, and new adventures and experiences. Within personality theory, this trait is found under somewhat different terms, but they cover essentially the same concept. In Costa and McCrae’s five-factor model, excitement seeking is a facet within the extraversion personality trait (Costa and McCrae, 1992; Buss, 1997; McCrae and Costa, 1997); in Eysenck’s three-factor model, sensation seeking is found within the extraversion trait (Eysenck and Eysenck, 1985; Matthews et al., 2003); and Zuckerman’s sensation seeking theory (Zuckerman, 1979, 1983, 1994) focuses only on sensation seeking. A person scoring high on these similar traits likes to be social with many people, seeks thrill and excitement, takes chances, is active until the point of restlessness, is optimistic and enthusiastic, and easily laughs and shows their feelings. More specifically, Zuckerman’s sensation seeking trait is defined as “the seeking of varied, novel, complex, and intense sensations and experiences, and the willingness to take physical, social, legal and financial risks for the sake of this experience” (Zuckerman, 1994).

Research indicates that these traits develop early in childhood, and individual differences in the urge for exploration, risk, and approaching new situations are found in groups as young as infants (Rothbart, 1988) and preschool-aged children (Morrongiello and Lasenby, 2006; Morrongiello et al., 2009, 2010). Studies show that children’s willingness to take risks is associated with their level of sensation seeking and how they perceive a risk situation, suggesting that children who are exhilarated by risks are more likely to engage in risky behavior and activities (Miller and Byrnes, 1997; Cook et al., 1999).

Studies of 3- to 8-year-old children’s temperament utilizing the Children’s Behavior Questionnaire (CBQ) have identified an extraversion/surgency factor, which is defined by high loadings on the labels of high-intensity pleasure, activity level, and impulsivity and a negative loading on shyness. In addition, positive anticipation and smiling and laughter are loaded considerably on the extraversion/surgency factor (Rothbart et al., 2000, 2001; Putnam et al., 2001). The extraversion/surgency dimension in temperament has been shown to exist in all age groups from 3 to 7 years old, and measures designed for even younger children (down to 3 months of age) show the existence of a similar extraversion/surgency dimension (Putnam et al., 2001).

These findings indicate a temperament dimension already existing in early childhood that resembles what is called excitement/sensation seeking and risk-taking personality among the adult population (Zuckerman, 1979, 1994; Horvath and Zuckerman, 1993; McCrae et al., 2000; Putnam et al., 2001). Children’s surgency in the CBQ is similar to the extraversion factor found in personality research in adults (Ahadi and Rothbart, 1994; Rothbart et al., 2001), and the surgency concept also appears to be closely related to Zuckerman’s (1994) sensation seeking trait. The high-intensity pleasure dimension in particular corresponds to the sensation seeking trait (Rothbart et al., 2001). Similarly, Strelau (1993) found that the activity dimension of temperament/personality was strongly correlated to Eysenck’s extraversion and Zuckerman’s sensation seeking traits in both young and adult populations. Relating to behavior, a high amount of extraversion/surgency in childhood would in general be apparent through high-intensity activities such as risky play and physical activity, a high activity level such as high levels of bodily movement, active indoor and outdoor play, impulsive behavior, and the absence of shyness (Putnam et al., 2001).

The most commonly used measure of sensation seeking among adults is Zuckerman’s Sensation Seeking Scale (SSS-V), which includes four subscales: Thrill and Adventure Seeking, Experience Seeking, Disinhibition, and Boredom Susceptibility (Zuckerman, 1994). The earliest versions of Zuckerman’s Sensation Seeking Scale (form I-IV) were developed based on several psychological theories within, e.g., optimal levels of stimulation and arousal, human drive theories, stimulus change and arousability theories, and inhibition theories (Zuckerman, 1979). SSS-V has been applied in research in many countries and cultural contexts, and all subscales have shown good cross-gender and cross-cultural replicability, except the Boredom Susceptibility subscale which has not been reproducible in all contexts (Zuckerman, 1994). Zuckerman (1994) has explained this to be a result of this subscale having more cultural specific items.

Since there are indications of a biological basis and heritability of the sensation seeking trait (see, e.g., Russo et al., 1991; Zuckerman, 1994) there has been an interest to study the existence of this personality trait also among young age groups. Measures for children’s sensation seeking have been explored throughout the last decades, but it has been deemed challenging to establish a suitable and robust measure. Russo et al. (1991) developed the Sensation Seeking Scale for Children (SSSC), based on Zuckerman’s sensation seeking scale but with modifications to fit children, and tested it in interviews of children aged 7–12 years. Factor analysis of Russo’s data showed two factors that corresponded to Boredom Susceptibility and Thrill and Adventure Seeking in Zuckerman’s scale for adults, as well as an adequate test–retest reliability. However, in a follow-up study, Russo et al. (1993) found three factors: Thrill and Adventure Seeking, Drug and Alcohol Attitudes, and Social Disinhibition. When using the SSSC, Morrongiello and Lasenby (2006) found that the focus on drugs, alcohol, and challenging social behavior was irrelevant for younger children, and they also found low reliability in the scale/subscales (α ranging from 0.40 to 0.50). They therefore developed a new sensation seeking scale for children (SSSC) that was better aligned to how such a trait might be expressed among 7- to 12-year-old children, focusing more on children’s activities and physical risk taking. The present study focuses on Morrongiello and Lasenby’s later version of SSSC. Morrongiello and Lasenby’s SSSC included five dimensions: Boredom Susceptibility (intolerance of monotonous, repetitive, and/or predictable people, tasks, and situations), Thrill Seeking (preferring arousing, exciting, and exhilarating activities), Behavioral Intensity (preferring intense, exciting, and emotionally arousing activities), Novel Experience Seeking (preferring new and unknown experiences more than familiar ones), and Inhibitory Control (impulsiveness and having a harder time controlling themselves and stopping a fun and rewarding activity). Morrongiello and Lasenby (2006) examined the criterion validity of the SSSC by comparing Canadian children’s SSSC scores with the results from mothers’ report of their children’s risk taking (a parent version of the SSSC and the Injury Behavior Checklist [IBC]) as well as children’s self-reported IBC, their intentions to take risks, and behavior in actual risk-taking tasks. The study revealed that the subscales Novelty Seeking (*α* = 0.32) and Boredom Susceptibility (*α* = 0.63) in the child-SSSC had no significant relation to child-SSSC or parent-SSSC, or to any of the other indices of risk taking, while Thrill Seeking and Behavioral Intensity were significantly related to all indices of risk taking. Behavioral Inhibition was related to all other indices of risk taking when reported by the children, but only to the IBC when parents were respondents. Tests of internal reliability showed that the child-SSSC subscales Thrill Seeking (*α* = 0.79), Behavioral Inhibition (*α* = 0.79), and Behavioral Intensity (*α* = 0.78) showed acceptable internal reliability, and that the subscales Novelty Seeking (*α* = 0.32) and Boredom Susceptibility (*α* = 0.63) failed to show acceptable internal reliability. Based on this, Morrongiello and Lasenby (2006) concluded that there is more variability in the parent-SSSC than the child-SSSC, and that in both versions the results indicated that three dimensions of sensation seeking is a better fit than five dimensions in studies looking at associations between sensation seeking and physical risk taking. The SSSC was later used in a new study with 102 Canadian children (7–12 years), where they report adequate internal reliability for Thrill Seeking (*α* = 0.78) and Behavioral Intensity (*α* = 0.75), although not for Behavioral Inhibition (labeled “Inhibitory Control” in that paper) (*α* = 0.66) (Morrongiello et al., 2009).

Building on the SSSC, Morrongiello et al. (2010) later developed a sensation seeking scale for young children (SSSYC) aged 2–5 years in which parents report on behalf of their children. The SSSYC included three subscales (Novelty Seeking, Thrill Seeking and Behavioral Intensity) as well as an Overall score. Results from the SSSYC was compared to children’s behavior during a free play task where sensation seeking behavior was coded, a Structured Sensation Seeking Activities task with activities matching each of the SSSYC subscales, and maternal reports on children’s past injuries to test evidence for criterion validity. Novelty Seeking was not related to any of the comparison measures, while Thrill Seeking, Behavioral Intensity and Overall score shoed significant correlations to children’s observed sensation seeking behavior during free play, and their history of minor and moderately severe injuries. The Overall score of the SSSYC correlated to the overall score of the Structured Sensation Seeking Activities task, and Thrill Seeking and Behavioral Intensity in SSSYC was positively related to the sum scores of the matching activities in the Structured Sensation Seeking Activities task. Novelty Seeking failed to correlate to the matching activities in the Structured Sensation Seeking Activities task. Tests of internal reliability scores were adequate for the Overall score (*α* = 0.84), Thrill Seeking (*α* = 0.86) and Behavioral Intensity (*α* = 0.82), but low for Novelty Seeking (*α* = 0.56). The SSSYC was later translated into German and used on a sample of 423 parents of 3- to 6-year-olds (Haas et al., 2019). That study did not test internal reliability of each of the three subscales, but the internal reliability of the overall scale was *α* = 0.76.

Another attempt to create a measure for children’s sensation seeking (9–13 years) was the development of the Brief Sensation Seeking Scale for Children (BSSS-C) by Jensen et al. (2011). This scale is a modification of a brief sensation seeking scale for adults, based on Zuckerman’s SSS-V and using four similar subscales. Analysis of the BSSS-C among US children showed that only one of the four factors was internally reliable (Thrill and Adventure Seeking, *α* = 0.82) in a sample of 9- to 13-year-olds (*M* = 10.87). Thus, Jensen et al. argued that the BSSS-C should be used uni-dimensionally, as the total measure of the scale had adequate internal reliability (*α* = 0.82) and showed evidence of criterion validity predicting other risky child behaviors such as video gaming, enjoyment of violent video games, and rule breaking. The BSSS-C (translated) was applied in a Dutch sample of 9- to 12-year-olds (*M* = 11.4), where the researchers reported evidence for criterion validity as BSSS-C scores predicted symptoms of attention problems and aggressive behavior. This study also found that the psychometric properties of the scale was good, and that internal reliability was adequate (*α* = 0.82) (Dekkers et al., 2019).

A number of studies on adult personality have reached the conclusion that men are more inclined toward sensation seeking than women (Zuckerman, 1979, 1994). This seems to be the case across cultures, such as the USA, England, Scotland, Japan, Thailand, Canada, Australia, and Spain (Zuckerman, 1994). Some studies have also found gender differences in children’s temperament/personality. Torgersen (1985) found that boys were higher on the temperament dimension “activity level” than girls at 6 years of age, but not in infancy, and Rothbart (1988) concluded that infant girls showed a higher amount of hesitation and inhibited approach than boys. Goldsmith et al. (1997) used several temperament measures and found that among toddlers and preschool-aged children, boys were higher than girls on “activity level” and “high pleasure,” while girls were higher than boys on “inhibitory control,” “perceptual sensitivity” and “effortful control.” Similarly, Goldberg (2001) found that teachers describe elementary school girls as less extroverted than boys. Focusing more particularly on children’s sensation seeking, several studies have found boys to score higher on this trait than girls among 9- to 13-year-olds (Jensen et al., 2011; Dekkers et al., 2019), and Thrill Seeking has been found to be higher for boys than girls among 3- to 6-year-olds (Haas et al., 2019).

Supporting the finding of gender differences in sensation seeking, research looking at children’s behavior and play preferences also indicates gender differences in the willingness to take risks and engage in risky play. Ginsburg and Miller (1982) found that boys were more willing to take risks than girls, and Smith (1998) found that more boys than girls participated in playground risk taking. Research has also found that boys engage in exceedingly more challenging physical play and rough-and-tumble play than girls (Blurton Jones, 1976; DiPietro, 1981; Humphreys and Smith, 1984, 1987; Eaton and Enns, 1986; Smith, 1997, 2005; Macdonald, 1998; Pellegrini and Smith, 1998; Power, 2000; Sandseter et al., 2020). This has also been confirmed in studies controlling for different maturational status between boys and girls (Eaton and Yu, 1989). Research findings also indicate that boys rate risk lower than girls (Hillier and Morrongiello, 1998), that boys are more likely than girls to be involved in “testing the limits” and risk-taking behaviors, and that boys are more likely to report having recently been physically injured (Cairns and Cairns, 1994).

Studies investigating age differences in sensation seeking and/or risk taking in childhood years have somewhat diverging results. On the one hand, Steinberg (2008) and Ellis et al. (2012) have suggested that risky behavior generally increases through childhood, peaks in adolescence, and decreases into adulthood. On the other hand, there are studies suggesting that risk preferences or risk taking decreases with typical age-related traits such as maturity (Andreoni et al., 2020) or behavioral inhibition (Edelson and Reyna, 2021). Lastly, there are studies exploring sensation seeking among 9- to 13-year-olds that find no age-related differences (Jensen et al., 2011; Dekkers et al., 2019). Thus, risk taking and sensation seeking are probably complex phenomena that are influenced by a variety of factors.

### 1.1 Aim of study

Based upon the presented considerations, it thus appears that subpopulations of children with different characteristics can report various tendencies for sensation seeking, which in turn can impact the validity of scales targeting this personality construct. Establishing such measurement invariance is highly important, as strong invariance is an important prerequisite for the consideration of mean subgroup comparisons, while strict invariance is a prerequisite for any form of between-group comparisons relying on manifest scale scores rather than latent factors (Meredith, 1993; Millsap, 2011). To the author’s knowledge, no previous research has examined the potential measurement invariance of the factor structure of the SSSC as a function of children’s gender and their corresponding differences in scores. The main aim of the current study was thus to examine the psychometric properties of a Norwegian translation of the SSSC in a sample of 7- to 10-year-old Norwegian children, investigating the scale’s internal consistency, factor structure, measurement invariance, and potential systematic response variations, considering the child’s age and gender.

## 2 Methods

The data analyzed in the present study were collected as a part of the Virtual Risk Management (ViRMa) project conducted among Norwegian 7- to 10-year-olds and their parents. The ViRMa study aims to examine children’s development of risk management skills through risky play. To accomplish this, the project aims to use and validate newly developed and ethically appropriate data collection tools such as virtual reality, eye tracking, and motion capturing and to provide insight into how children assess and handle risk situations, as well as how children’s past risky play experiences are associated with their risk management (Sandseter et al., 2023). To include children’s propensity to seek risk in the analysis in the ViRMa study, the SSSC was included as a data collection tool. The Norwegian Data Protection Services for Research (ref. no. 784782) approved the study.

### 2.1 Participants

Participants were recruited from four primary schools in Norway (grades 2–4). Schools were recruited by the researchers and varied in size (from around 240 pupils to 580 pupils) and geographical location (i.e., city or village schools). The inclusion criteria were outlined as follows: (1) Age ranged between 7 and 10 years (corresponding to grades 2, 3, and 4 in Norwegian schools), (2) Informed consent was obtained from parents for participation, and (3) Assent was obtained from the child for participation. We initiated contact with selected schools through the principal, seeking their willingness to participate. Upon agreement, parents were subsequently reached through the school’s web-based information system, with information about the project and an electronic consent form. Children whose parents granted consent were added to a participant list, with names replaced by a random code. Children were also asked for assent to participate, and in case of refusal, their name and code were deleted. Both parents and children were informed that they could withdraw from the study at any time throughout the project and have their data removed from the study. The sample in this study consists of 393 children, including 199 girls and 194 boys, who completed all questions in the SSSC questionnaire. The average age of these children was 8.8 years (SD = 0.9), with ages ranging from 7.2 to 10.6 years.

### 2.2 Measure

The translation and adaptation of the SSSC was performed according to Gudmundsson’s (2009) guidelines for translating and adapting psychological instruments; (1) selecting an instrument for translation (considering reliability and validity in the original instrument), (2) norms and standardization sample (reviewing the psychometric propertied in the original instrument), (3) selecting qualified experts (for doing the translation and adaptation), (4) method of translation (either translation and backtranslation, or translation of two independent experts and then comparison of the two by a third-party), (5) method of adaptation (considering adaptation of items to fit the new context/country), (6) investigating bias (considering construct bias, item bias and method bias), (7) pilot studies (first as small pilot study to assess wording and meaning of items, scoring and administration, and then a second pilot to evaluate the changes in the first pilot), (8) validity study (validity study in the target language to test factor structure and psychometric properties). In this study, the SSSC was selected because it is the only sensation seeking scale available for the age group in focus, and it has shown promising results of reliability and criterion validity (Morrongiello and Lasenby, 2006). The researchers in this project have worked with research on children’s risk-taking, risky play, sensation seeking personality and relevant statistics and data processing over a number of years and can be considered qualified experts. The SSSC was translated from English into Norwegian by one of the researchers in the project. All primary researchers in the project group discussed the translation to revise unclear wordings and adapt words and phrases to fit the Norwegian context. The Norwegian version was back-translated into English by a professional translator (Norwegian citizen living and working in the United States), and then the three versions (original English, translated Norwegian, and back-translated English) were discussed among the project group to ensure that the Norwegian version reflected the original meaning of the statements and was relevant and understandable for Norwegian children. The first adapted version of the SSSC was piloted in a small sample of 7 children (5 girls and 2 boys, aged 7–12 years) to assess if the wording and meaning of items was relevant to the age group. Some minor adjustments of wording were made to some of the items. Then a larger pilot was conducted with 64 children from grade 2, 3, and 4 (7-10-year-olds) in a Norwegian elementary school (37 boys, 27 girls), which showed that the scale was feasible for Norwegian children in this age group. This paper reports on the validity study of the final Norwegian version of SSSC with a larger (*n* = 393) sample of 7-10-year-old children.

The SSSC consists of 27 items, each presenting two statements of preference that children can choose between. For example, children are presented the following options:

*I am the sort of person who would like to sled fast down a steep hill.*

*OR*

*Sledding fast down a steep hill sounds scary to me.*

*Sailing on the ocean in a small boat would be dangerous and foolish.*

*OR*

*I think it would be fun to sail on the ocean in a small boat.*

*I would sometime like to try jumping from a plane with a parachute.*

*OR*

*I would never try jumping from a plane with a parachute.*

Selecting the statement indicating higher levels of the measured subscale earns 1 point on the overall subscale score. In the first and third example above, selecting the first option earns 1 point on the Thrill Seeking subscale, whereas choosing the second statement earns 0 points; while in the second example the child earn 1 point for choosing the second statement. In this study, we chose to include the three SSSC subscales that were found to be relevant to physical risk taking in earlier research (Morrongiello and Lasenby, 2006): Thrill Seeking, Behavioral Inhibition, and Behavioral Intensity.

### 2.3 Procedure

The SSSC was administered orally in an interview between one of the researchers in the project and the participating child, to prevent potentially biases in reading skills because many of the participating children were not fluent in reading (children start learning to read at 6 years of age). All children spoke Norwegian fluently and therefore language barriers were not a problem. The children received the same oral information prior to the interview (that we would ask them some questions about things they like/do not like to do, and things they would like/not like to try someday), and the questions were read to the child as they are worded in the questionnaire, and in a neutral way. The researcher recorded the child’s responses in a digital questionnaire stored on a computer. The interview was conducted at school, during school hours, with one child at a time in a group room or a secluded space where the child and researcher would not be disturbed.

### 2.4 Data analysis

Descriptive analysis was conducted to report the responses to each of the statements by gender. To evaluate the internal consistency and factor structure of the model proposed by Morrongiello and Lasenby (2006), Cronbach’s alpha, inter-item correlations, and confirmatory factor analysis (CFA) (Brown, 2014) were used. All CFA analyses used the maximum likelihood with robust standard errors to account for the dichotomous items. We evaluated the proposed models based on several commonly used criteria for model fit, while remaining open to various levels of acceptability. These criteria included RMSEA (< 0.1), SRMR (<0.1), CFI (>0.9), and TLI (>0.9), following the guidelines outlined by Mehmetoglu and Jakobsen (2017). In addition to these traditional fit indices, we also considered *R*^2^ estimates (with a general guideline of >0.25) and standardized factor loadings (with a general guideline of >0.40) when examining the factor loadings (Brown, 2014). If the proposed theoretical model was found to not meet the specified acceptable model fit indices, alternative models were explored using exploratory factor analysis (EFA) (Brown, 2014).

The influence of gender and age on the factor structures within the models was examined by multiple-group CFA invariance evaluation (Brown, 2014) to determine whether the factor structure remained consistent across different groups. This analysis enabled the assessment of whether the relationships between the observed indicators and the latent factors are comparable and invariant across various groups. In this study, the following analyses of measurement invariance were performed: (1) equal form, (2) equal factor loadings, (3) equal intercepts, (4) equal indicator residual variances, and (5) equal factor means (Brown, 2014). Stata/MP version 18 (StataCorp) was used for all statistical analyses.

## 3 Results

The mean of the sum scores of the 11 items related to the subscale Thrill Seeking was 6.7 (SD = 3.0), ranging from 0 to 11. The sum score of the seven items indicating Behavioral Intensity was 4.7 (SD = 1.9), ranging from 0 to 7. Behavioral Inhibition contained nine items with a sum score of 6.4 (SD = 2.0), ranging from 1 to 9. Table 1 presents descriptive statistics and the rating for each item by gender.

**Table 1:** Percentage of boys and girls refuting and confirming each statement and Discrimination Index (DI) for each item (*n* = 393).

Subscales and items	DI	Refuted (%)		Confirmed (%)		Girls	Boys	Girls	Boys
Thrill seeking					
Jump or dive of diving board	0.33	39	28	61	72
Try water-ski	0.25	43	32	57	68
Tricks and new things when riding a bike	−0.08	65	43	35	57
Swim in deep water	0.33	33	34	67	66
Jumping from a plane with a parachute	0.02	54	44	46	56
Like to do things that are a little scary	0.58	27	14	73	86
Try mountain climbing	0.02	47	51	53	49
Sail on the ocean in a small boat	0.15	51	34	49	66
Try fun things that are unknown and scary	0.50	30	20	70	80
Try surf-board riding	0.32	42	26	58	74
Fun to ski fast down a snowy mountain	−0.06	64	42	36	58
Behavioral intensity					
Sledge fast down a steep hill	0.58	31	11	69	89
Ride bike very fast down steep hill	0.05	60	34	40	66
Climb to the top of a tall ladder	0.38	36	26	64	74
Like places with large crowds and excitement	0.32	39	29	61	71
Would like to go on vacation to an exotic place	0.55	23	22	77	78
Exciting to do risky sports	0.12	48	40	52	60
Like roller-coasters and other fast rides	0.45	28	27	71	73
Behavioral inhibition					
Wait my turn before answering in class	0.65	8	27	92	73
Stop myself from fun things that I should not do	0.34	30	36	70	64
When having a good time I do not mind leaving	0.08	45	47	55	53
Finish tasks once I make up my mind about it	0.48	21	31	79	69
Easy for me to stop enjoyable things when told	0.17	41	43	59	57
Think things through before I speak	0.55	20	25	80	75
I do not peak at presents before I get them	0.54	23	23	77	77
Keeping secrets is easy for me	0.76	16	9	84	91
It is not hard to wait for my turn when playing	0.29	33	38	67	62

To determine the internal consistency reliability of the three subscales within the proposed theoretical model, Cronbach’s alpha was used. The obtained Cronbach’s alpha coefficient for Thrill Seeking (*α* = 0.79) was above the commonly accepted threshold of 0.7, while the coefficients for Behavioral Intensity (*α* = 0.66) and Behavioral Inhibition (*α* = 0.64) were below the threshold. Average inter-item correlations were conducted to further evaluate the internal consistency and homogeneity. The average inter-item correlation was lower than 0.20 for the subscale Behavioral Inhibition (0.16), while Thrill Seeking (0.26) and Behavioral Intensity (0.22) were within the ideal range (between 0.20 and 0.40) of average inter-item correlation. These results indicate questionable internal consistency for the Behavioral Intensity and Behavioral Inhibition subscales.

Next, the proposed model was evaluated using confirmatory factor analysis (CFA). The exogenous latent variables were allowed to be correlated, and Thrill Seeking was closely associated with Behavioral Intensity (*β* = 0.95, *p* < 0.001). The squared correlation between Thrill Seeking and Behavioral Intensity was also high (0.90), suggesting a substantial shared variance between these constructs. Behavioral Inhibition was not significantly associated to Thrill Seeking and Behavioral Intensity. The model fit measures for the model suggested an acceptable fit to the data (*x*^2^ = 507 [321], RMSEA = 0.038, SRMR = 0.053, CFI = 0.89, TLI = 0.88). The chi-square value indicated a statistically significant deviation between the observed and expected values based on the specified model. However, the chi-square test is sensitive to sample size, and with larger samples, even minor deviations can result in a significant chi-square value. The other indices suggested an acceptable fit of the model.

Following the questionable internal consistency and possible overlap between Thrill Seeking and Behavioral Intensity, an exploratory factor analysis (EFA) was conducted to address the issue and explore an alternative measurement model for the items. Initially, a principal factor analysis revealed two factors with eigenvalues above 1.0. Additional analyses were conducted to confirm a two-factor solution’s appropriateness, including a scree plot examination, assessment of extracted variance, and parallel analysis. The two factors were interpreted as follows: factor one (Thrill and Intensity Seeking) was considered to be related to Thrill Seeking and Intensity Seeking since all items with substantial factor loadings came from the Thrill Seeking and Behavioral Intensity subscales, while factor two consisted of items from the Behavioral Inhibition subscale and thus kept this name. Table 2 presents the two selected factors’ rotated factor loadings (orthogonal varimax).

**Table 2:** Rotated factor loadings and uniqueness for selected factors (*n* = 393).

Items	Factor one	Factor two	Uniqueness
Jump or dive of diving board	0.241	−0.029	0.571
Try water-ski	**0.375**	−0.010	0.542
Tricks and new things when riding a bike	**0.642**	−0.071	0.549
Swim in deep water	0.128	0.041	0.682
Jumping from a plane with a parachute	**0.494**	−0.049	0.610
Like to do things that are a little scary	**0.389**	0.028	0.609
Try mountain climbing	**0.359**	−0.047	0.607
Sail on the ocean in a small boat	**0.322**	0.031	0.753
Try fun things that are unknown and scary	0.287	0.035	0.645
Try surf-board riding	**0.420**	0.032	0.560
Fun to ski fast down a snowy mountain	**0.448**	−0.012	0.635
Sledge fast down a steep hill	**0.328**	−0.004	0.586
Ride bike very fast down steep hill	**0.504**	0.002	0.576
Climb to the top of a tall ladder	**0.327**	−0.004	0.628
Like places with large crowds and excitement	0.188	0.021	0.803
Would like to go on vacation to an exotic place	**0.333**	0.008	0.763
Exciting to do risky sports	**0.643**	0.014	0.522
Like roller-coasters and other fast rides	0.273	−0.020	0.717
Wait my turn before answering in class	−0.139	**0.511**	0.631
Stop myself from fun things that I should not do	0.006	**0.485**	0.717
When having a good time I do not mind leaving	0.037	**0.390**	0.772
Finish tasks once I make up my mind about it	−0.029	**0.315**	0.759
Easy for me to stop enjoyable things when told	0.005	**0.604**	0.627
Think things through before I speak	−0.028	**0.300**	0.793
I do not peak at presents before I get them	−0.044	0.235	0.826
Keeping secrets is easy for me	0.015	0.175	0.837
It is not hard to wait for my turn when playing	−0.004	**0.512**	0.691

Factor loadings > 0.30 are bolded.

To choose the items for further analysis, a relatively low threshold of 0.30 was set for rotated factor loadings. This was done intentionally to include various activities in the measurement model to capture the multidimensionality of the constructs. Based on this criterion, three items from the Thrill Seeking subscale (diving board, deep water, and fun and scary things), two items from the Behavioral Intensity subscale (large crowds and roller coasters), and two items from the Behavioral Inhibition subscale (presents and secrets) were excluded from further analysis. Ultimately, the factor analysis process resulted in a two-factor solution consisting of Thrill and Intensity Seeking (13 items) and Behavioral Inhibition (7 items). As discussed below, these factors were further analyzed for internal consistency, model fit, and model invariance.

Analysis of Cronbach’s alpha demonstrated good internal consistency for Thrill and Intensity Seeking (*α* = 0.83) and low internal consistency for Behavioral Inhibition (*α* = 0.65). The average inter-item correlation was acceptable for both Thrill and Intensity Seeking (0.27) and Behavioral Inhibition (0.21). Next, the proposed model was examined using confirmatory factor analysis (CFA). The exogenous latent variables were allowed to be correlated, and Thrill and Intensity Seeking and Behavioral Inhibition were unrelated (*β* = −0.08, *p* = 0.238). Boys (girl = 0, boy = 1) scored significantly higher on the latent variable Thrill and Intensity Seeking (*β* = 0.26, *p* < 0.001) and significantly lower on the Behavioral Inhibition construct (*β* = −0.18, *p* = 0.004). Age was positively related to both Thrill and Intensity Seeking (*β* = 0.21, p < 0.001) and Behavioral Inhibition (*β* = 0.12, *p* = 0.043). The standardized factor loading in the measurement model (Table 3) for Thrill and Intensity Seeking was between 0.62 and 0.34, and *R*^2^ estimates ranged from 0.38 to 0.12. In the Behavioral Inhibition construct, the standardized factor loadings were between 0.64 and 0.32, and the *R*^2^ estimates between 0.41 and 0.10.

**Table 3:** Standardized factor loadings (λ) and *R*^2^ estimates from the 2-Factor Solution of the SSSC using CFA (*n* = 393).

Items	Thrill and intensity seeking		Behavioral inhibition		λ	*R* ^2^	λ	*R* ^2^
Try water-ski	0.58	0.34		
Tricks and new things when riding bike	0.62	0.38		
Jumping from plane with parachute	0.58	0.33		
Like to do things that are a little scary	0.49	0.24		
Try mountain climbing	0.53	0.28		
Sail on the ocean in a small boat	0.38	0.15		
Try surf-board riding	0.56	0.32		
Fun to ski fast down a snowy mountain	0.53	0.28		
Sled fast down a steep hill	0.47	0.22		
Ride bike very fast down steep hill	0.56	0.31		
Climb to the top of a tall ladder	0.46	0.21		
Would like to go on vacation to exotic place	0.34	0.12		
Exciting to do risky sports	0.64	0.41		
Wait my turn before answering in class			0.53	0.28
Stop myself from fun things that I should not do			0.48	0.23
When having a good time I do not mind leaving			0.40	0.16
Finish tasks once I make up my mind about it			0.33	0.11
Easy for me to stop enjoyable things when told			0.64	0.41
Think things through before I speak			0.32	0.10
It is not hard to wait for my turn when playing			0.49	0.24

The model fit measures (Table 4) for the alternative two-factor model indicated a relatively good model fit (*x*^2^ = 234 [169], RMSEA = 0.038, SRMR = 0.050, CFI = 0.93, TLI = 0.92). While the chi-square test showed a significant deviation between the observed and expected values based on the specified model, the CFI and TLI indicated a good fit. In addition, RMSEA and SRMR supported the notion that the model is a good fit. The alternative two-factor model was used for invariance testing based on these results.

**Table 4:** The Goodness-of-Fit Indexes of the CFA models for the SSSC.

Model	*N*	χ^2^ (df)	CFI	TLI	RMSEA	SRMR
Three-factor model	393	507 (321)	0.89	0.88	0.038	0.053
Two-factor model	393	264 (169)	0.93	0.92	0.038	0.050
Two-factor model boys	194	238 (169)	0.90	0.89	0.046	0.065
Two-factor model girls	199	212 (169)	0.92	0.91	0.036	0.063
Two-factor model younger	195	203 (169)	0.95	0.94	0.032	0.060
Two-factor model older	201	222 (169)	0.92	0.91	0.040	0.062

χ^2^, chi-square; df, degrees of freedom; CFI, comparative fit index; RMSEA, Root Mean Square Error of Approximation; SRMR, standardized root mean residual; TLI, Tucker-Lewis index. Probability level *p* < 0.05.

We conducted a multi-group confirmatory factor analysis (CFA) using the proposed two-factor theoretical model to examine measurement invariance for the factor structure across gender and age. Initially, separate models were fitted for each group, as presented in Table 4. Although the fit indices suggested a slightly better fit for girls than for boys, these results indicated that the two-factor model provides a comparable fit to the full sample-factor model across different groups, including boys and girls and younger and older participants in the present sample. To further examine measurement invariance, subsequent tests were conducted. For boys and girls, the results revealed significant differences in factor loadings (χ^2^diff = 42.8 [18], *p* < 0.001), intercepts (χ^2^diff = 79.9 [18], *p* < 0.001), error variances (χ^2^diff = 75.1 [20], *p* < 0.001), and factor means (χ^2^diff = 33.6 [2], *p* < 0.001) between boys and girls. The tests for the two age groups demonstrated no significant differences in factor loadings, intercepts, or error variances. However, factor means were found to be significantly different between older and younger children (χ^2^diff = 18.5 [2], *p* < 0.001). These findings indicate that the measurement properties of the two-factor model significantly differ between boys and girls and that the two age groups in the present sample had a limited impact on the measurement model.

## 4 Discussion

The main aim of this study was to examine the psychometric properties of a Norwegian translation of the SSSC in a sample of 7- to 10-year-old Norwegian children, investigating the scale’s internal consistency, factor structure, measurement invariance, and potential systematic response variations, considering the child’s age and gender.

Overall, the results showed that we were not able to replicate the exact same subscales that Morrongiello and Lasenby (2006) found in their analysis of the English version, tested with Canadian children. While Morrongiello and Lasenby concluded that children’s sensation seeking comprises three dimensions—Thrill Seeking, Behavioral Inhibition, and Behavioral Intensity—with acceptable internal reliability, our analysis of the Norwegian version tested with Norwegian children failed to show acceptable internal reliability for Behavioral Intensity and Behavioral Inhibition. Further CFA showed that Thrill Seeking was highly associated with and somewhat overlapping with Behavioral Intensity, and the EFA revealed that a two-factor structure gave the best fit of our data: (1) a combination between Thrill Seeking and Behavioral Intensity, which we renamed Thrill and Intensity Seeking, and (2) Behavioral Inhibition. In both factors, some of the original items from the SSSC were removed due to low factor loadings. A CFA for the two factors using the remaining items showed a relatively good model fit. Thrill and Intensity Seeking and Behavioral Inhibition were also shown not to be correlated, indicating that two different dimensions of sensation seeking are captured by the model.

Considering our findings, there is a theoretical logic to the overlap between Thrill Seeking and Behavioral Intensity in the original SSSC. The Thrill Seeking subscale has questions about whether or not a child would like to try mountain climbing, waterskiing, jumping from a plane with a parachute, etc. The Behavioral Intensity subscale includes questions such as whether the child would like to sled fast down a steep hill, climb to the top of a tall ladder, do risky sports, etc. (Morrongiello and Lasenby, 2006). Even though the questions in the Behavioral Intensity scale indicate a higher intensity in the activity, with words such as “fast,” “steep,” “tall,” and “risky,” most of the activities mentioned are similar to items in Zuckerman’s (1994) Thrill and Adventure Seeking, which covers finding pleasure in participating in adventurous activities and risky experiences that provide intense sensations (e.g., rock climbing, sky diving, or river kayaking). In Zuckerman’s Thrill and Adventure Seeking subscale, there is a mix of items with and without indications of intensity, e.g., *I would like to try parachute jumping* and *I think I would enjoy the sensations of skiing very fast down a high mountain slope* (Zuckerman, 1994). In this perspective, it is not a surprise that children’s answers to these two subscales from the SSSC are highly correlated and seem to measure the same dimension.

In line with the study of Haas et al. (2019) of 3- to 6-year-olds, our analysis of the Norwegian version of the SSSC and the new subscales developed in this study shows that boys score higher than girls on Thrill and Intensity Seeking. This supports findings of boys being higher than girls on temperament dimensions such as “activity level” (Torgersen, 1985; Goldsmith et al., 1997). It also supports studies showing that relative to girls, boys’ behavior is more characterized by the willingness to take risks (Ginsburg and Miller, 1982; Smith, 1998) and by challenging physical play and rough-and-tumble play (Blurton Jones, 1976; DiPietro, 1981; Humphreys and Smith, 1984, 1987; Eaton and Enns, 1986; Smith, 1997, 2005; Macdonald, 1998; Pellegrini and Smith, 1998; Power, 2000; Sandseter et al., 2020). In the present study, boys also scored significantly lower than girls on Behavioral Inhibition. This is in alignment with earlier findings showing that girls are more hesitant, have more inhibited and effortful control, and are less extroverted than boys (Rothbart, 1988; Goldsmith et al., 1997; Goldberg, 2001).

In the two-factor solution in our sample, the results show that age is positively related to both Thrill and Intensity Seeking and Behavioral Inhibition, meaning that older children tend to score higher on both subscales. Although earlier studies have shown diverging results on sensation-seeking and risk-taking behavior in relation to age, the theoretical assumption is that such behavior increases during childhood toward a peak in adolescence (between 19 and 24 years) (Zuckerman, 1994; Steinberg, 2008; Ellis et al., 2012). The increasing level of Thrill and Intensity Seeking with age in our study would support such an assumption, even though it is worth noting that the span of ages in our study is limited to only 3 years (ages 7–10). Studies on similar age groups, such as Jensen et al. (2011) and Dekkers et al. (2019) studying 9- to 13-year-olds, have not been able to find age-related differences. The observed positive relationship between age and Behavioral Inhibition suggests that older children tend to exhibit slightly greater self-control in comparison to their younger counterparts. However, it is important to note that the strength of this association is relatively weak and that the statistical significance, while present, is just below the conventional threshold of 0.05. Consequently, the practical implications of this finding are considered to be somewhat limited. This result is in line with the expectation that older pupils, having spent more time in the school environment, may show a higher inclination to respond affirmatively to statements like *I wait my turn before answering in class*. Their increased exposure to classroom norms and routines may contribute to a modest increase in their Behavioral Inhibition scores. This could also be an expression of compliance with social norms, where the children merely answer what is socially accepted (e.g., to wait one’s turn) in the test situation, rather than what they actually do in class. Nevertheless, it is essential to recognize that the observed effect is not substantial, and a range of other factors likely contribute to individual differences in Behavioral Inhibition.

The current study focused on evaluating the psychometric properties of a Norwegian translation of the SSSC within a sample of 7- to 10-year-old Norwegian children. Despite the valuable insights gained, several limitations in the methodology of this study must be considered. First, this study did not include an assessment of the test–retest reliability or aspects of validity such as convergence with similar questionnaires or whether the Norwegian version of the SSSC can predict scores on other psychological measures. Second, the study design was cross-sectional, capturing a single point in time, which limits the ability to explore the consistency of the measure across time. Thirdly, it is important to acknowledge that the study relied on a convenience sample of 393 children recruited from four primary schools in Norway. The schools varied in size and geographical location, adding some heterogeneity to the sample. However, the convenience sampling approach might not fully capture the diversity within the population of Norwegian children.

Furthermore, it is important to acknowledge that the SSSC was initially developed in a broader age range, from 7 to 12 years. While the SSSC has demonstrated its utility in this wider age range, our study focused on a specific subset of this range, encompassing 7- to 10-year-old Norwegian children. This age restriction may have implications for the generalizability of our findings to the entire intended age range of the scale. Researchers should consider potential age-related variations when interpreting the results within the 7- to 10-year-old subset. While our study aimed to evaluate measurement invariance through Confirmatory Factor Analysis (CFA) following an Exploratory Factor Analysis (EFA), it is important to note that the potential for overfitting and capitalization on chance exists. Future research with more diverse samples and extensive age ranges could provide a more comprehensive understanding of the scale’s performance and generalizability. There are also social and cultural aspects of sensation seeking that might influence the results, especially when children self-report and in a test situation where an adult asks the questions. Comparisons with observations could be a way forward, for example by observing practical tasks or situation where children can play unsupervised with equipment or in environments that afford risk taking similar to what Morrongiello et al. (2010) did with younger children.

In summary, our findings suggests that the SSSC, as it was developed in a Canadian context and with Canadian children, does not transfer directly to the Norwegian context. In our study, sensation seeking consisted of two factors, instead of the original three. However, gender differences were replicated, with boys scoring higher on Thrill and Intensity Seeking and girls scoring higher on Behavioral Inhibition. The positive relationships between age and the two dimensions of sensation seeking were in line with some previous research but remain difficult to interpret. Future applications of the SSSC should consider these aspects, including issues related to social dynamics and children’s self-reporting.

## Data availability statement

The raw data supporting the conclusions of this article will be made available by the authors, without undue reservation.

## Ethics statement

The studies involving humans were approved by The Norwegian Data Protection Services for Research (ref. no. 784782). The studies were conducted in accordance with the local legislation and institutional requirements. Written informed consent for participation in this study was provided by the participants’ legal guardians/next of kin.

## Author contributions

ES: Conceptualization, Funding acquisition, Investigation, Methodology, Project administration, Writing – original draft, Writing – review & editing. OS: Conceptualization, Data curation, Formal analysis, Investigation, Methodology, Writing – review & editing. RK: Conceptualization, Investigation, Methodology, Writing – review & editing. HL: Investigation, Methodology, Writing – review & editing. LS: Investigation, Writing – review & editing.
